# A Narrative Review of COVID-19 Vaccines

**DOI:** 10.3390/vaccines10010062

**Published:** 2021-12-31

**Authors:** Barbaros Eroglu, Rina Fajri Nuwarda, Iqbal Ramzan, Veysel Kayser

**Affiliations:** Sydney Pharmacy School, Faculty of Medicine and Health, The University of Sydney, Sydney 2006, Australia; barbaros.eroglu@sydney.edu.au (B.E.); rnuw0385@uni.sydney.edu.au (R.F.N.); iqbal.ramzan@sydney.edu.au (I.R.)

**Keywords:** COVID-19, COVID-19 vaccines, SARS-CoV-2, vaccination, vaccine hesitancy

## Abstract

The COVID-19 pandemic has shaken the world since early 2020 and its health, social, economic, and societal negative impacts at the global scale have been catastrophic. Since the early days of the pandemic, development of safe and effective vaccines was judged to be the best possible tool to minimize the effects of this pandemic. Drastic public health measures were put into place to stop the spread of the virus, with the hope that vaccines would be available soon. Thanks to the extraordinary commitments of many organizations and individuals from around the globe and the collaborative effort of many international scientists, vaccines against COVID-19 received regulatory approval for emergency human use in many jurisdictions in less than a year after the identification of the viral sequence. Several of these vaccines have been in use for some time; however, the pandemic is still ongoing and likely to persist for the foreseeable future. This is due to many reasons including reduced compliance with public health restrictions, limited vaccine manufacturing/distribution capacity, high rates of vaccine hesitancy, and the emergence of new variants with the capacity to spread more easily and to evade current vaccines. Here we discuss the discovery and availability of COVID-19 vaccines and evolving issues around mass vaccination programs.

## 1. Introduction

In December 2019, Chinese authorities notified the World Health Organization (WHO) of a cluster of unusual pneumonia cases in Wuhan, of an unknown viral aetiology. The cause of the cases was subsequently identified as a new type of coronavirus, later named Severe Acute Respiratory Syndrome Coronavirus 2 (SARS-CoV-2), and the disease caused by this virus was named Coronavirus Disease 2019 (COVID-19) [1]. In March 2020, WHO declared the COVID-19 outbreak a pandemic and warned governments globally to take all the necessary measures to prevent its spread [2]. Due to its high transmission rates, governments and public health agencies worldwide adopted public awareness campaigns and enforced social distancing restrictions, to minimize person-to-person transmission of the virus. Despite many efforts to contain the spread of the disease, including mandatory face mask wearing in public places, cancelling or limiting the number of people at gatherings and implementing lockdowns in many countries, the overall number of COVID-19 cases has exceeded 250 million worldwide with over 5.1 million deaths as of 29 November 2021 [3]. The severity of the pandemic led many scientists worldwide to focus on finding ways to treat the disease. Discovering and developing vaccines to fight COVID-19 has been the main objective of researchers and pharmaceutical companies. This review discusses the key elements of the SARS-CoV-2, COVID-19 disease, vaccines that have been developed very rapidly and are now commercially available and the pipeline of new vaccine candidates that are yet to enter the commercial arena. Even after two years, the COVID-19 pandemic continues as a major public health concern. Thus, providing a contemporary review of currently available and emerging COVID-19 vaccines globally would be a useful addition to the literature on the COVID-19 pandemic.

Key topics that are included in this review include:

Background on COVID-19 pandemic as well as on beta-coronaviruses more generally, clinical overview of COVID-19, traditional and contemporary vaccine discovery platforms, classes of COVID-19 vaccines and key current and emerging COVID-19 vaccination themes like universal availability and affordability, vaccine hesitancy and the outlook for novel COVID-19 vaccines to tackle emerging variants.

This is not the first instance in the 21st century that a new and dangerous coronavirus has made the headlines. The first time that viruses from the coronavirus family shocked the world was in 2003 when a beta-coronavirus (SARS-CoV) caused an outbreak in China and spread to four other countries, causing more than 700 deaths with around 50% mortality in individuals over the age of 60 [4]. In 2012, another coronavirus, MERS-CoV, was identified causing Middle East respiratory syndrome (MERS), a deadly respiratory infection with a mortality rate of approximately 40% [5]. Even though SARS-CoV-2 is not as deadly as previous coronaviruses, it spreads far more efficiently than previous coronaviruses and has become a major global public health concern in only a couple of months.

**Figure 1:**
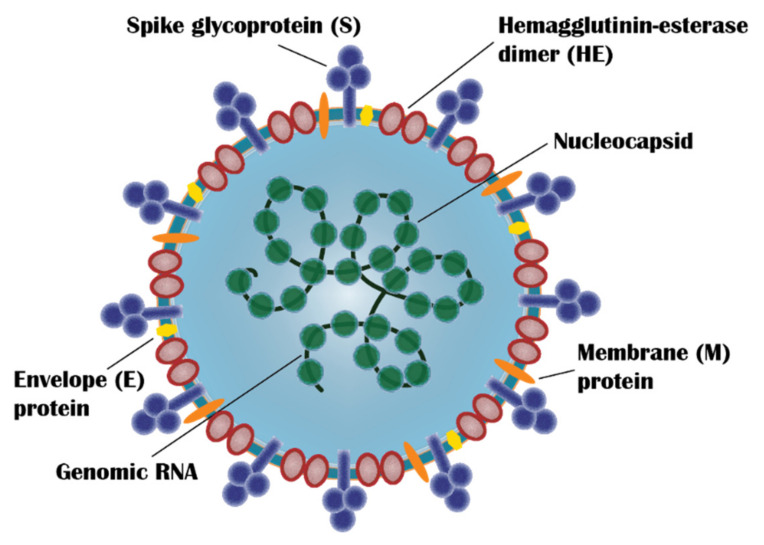
Schematic drawing of SARS-CoV-2 representing the major virus components: spike glycoprotein (S), hemagglutinin-esterase dimer (HE), membrane protein (M), envelope protein (E), nucleocapsid, and genomic RNA. (Redrawn by the authors based on Figure 2 in Ref. [13]).

SARS-CoV-2 is a member of the coronavirus family and belongs to the genera, beta-coronaviruses. It shares a similar genome sequence with other well-known beta-coronaviruses, SARS-CoV (~79%) and MERS-CoV (~50%) [6]. It is a single-stranded RNA virus with a genome size of ~30 kb and expresses 29 proteins including four main SARS-CoV-2 proteins: Spike (S) protein, Envelope (E) protein, Membrane (M) protein and Nucleocapsid (N) protein [7] (Figure 1). S protein is the major surface protein of SARS-CoV-2 and is responsible for cell entry [8]. It is a 1273 amino acid trimeric protein with each monomer consisting of two subunits, S1 and S2 [9]. S1 subunit (aa14-685) consists of N-terminal domain, Receptor Binding Domain (RBD) and receptor-binding motif and facilitates the binding of the virus to the host cell receptor. S2 subunit (aa686-1273) comprises of fusion peptide, heptad repat 1 (HR1), heptad repeat 2 (HR2), transmembrane domain and cytoplasm domain, and mediates membrane fusion and entry into the host cell [10,11]. Structurally, S protein is classified as a class 1 viral fusion protein due to its characteristic heptad repeat region and N-terminal or N-proximal fusion peptide and formation of a heterotrimeric six-helix bundle during the membrane fusion process [8]. S protein has also been shown to generate strong immune response and elicit neutralizing antibodies which has led vaccine developers to focus on utilizing not only the S protein itself but also parts of the protein that trigger the immune response [12].

## 2. SARS-CoV-2 Variants

Mutation, alterations in one or several amino acids in the virus genome, can arise as a product of viral replication or natural selection. Although most of the amino acid changes are expected to be insignificant, some mutations yield differences in antigenicity, transmissibility, or virulence, resulting in new viral variants [14]. Throughout the COVID-19 pandemic, the emergence of new SARS-CoV-2 variants has been observed. The USA Centers for Disease Control and Prevention (CDC) has classified these as variants of interest, variants of concern, and variants of high consequence [15].

Variants that are predicted to impact the transmission, diagnostics, treatments, or immune evasion and are responsible for an increasing number of outbreaks are classified as variants of interest. Such a variant of interest may necessitate one or more additional public health actions, such as improved laboratory characterization, increased sequence surveillance, or epidemiological investigations to determine how easily the virus spreads to others, the severity of disease, therapeutic efficacy, and whether currently approved vaccines provide protection. A variant of interest (VOI) is classified as a variant of concern (VOC) if there is evidence for surge in transmissibility, increase in disease severity, and decreased efficacy of therapeutics or vaccines, or failure in its diagnosis. Variants of concern may require escalation of one or more public health responses, such as notification to WHO, efforts to control spread, increased testing, or studies to determine the efficacy of vaccines and treatments against the variant. Additional considerations such as developing new diagnostic tools or modifying treatments or vaccines may be necessary depending on the variant’s characteristics. Compared to previously circulating variants, a variant of high concern has clear evidence that preventative efforts or medical countermeasures (MCMs) are much less effective [15].

As of November 2021, six previous VOI (Epsilon, Eta, Iota, Kappa, Zeta, and Lambda) and three previous VOC (Alpha, Beta, and Gamma) have been designated as Variants Being Monitored (VBM) by CDC. VOI or VOC may be moved to this list if its national and regional prevalence has decreased significantly and sustainably over time, or if other evidence suggests that the variant does not pose a major risk to public health. Mu variant, which was detected in Columbia in January 2021, was designated as VBM in September 2021; no SARS-CoV-2 variants are currently considered as being of high consequences and variants of interest (Table 1). On 26 November 2021, The Technical Advisory Group on SARS-CoV-2 Virus Evolution (TAG-VE) was convened to evaluate a newly detected the SARS-CoV-2 variant B.1.1.529. This variant was designated as a VOC, named Omicron, first reported in South Africa on 24 November 2021. The first confirmed infection with the Omicron variant was identified in a specimen obtained on 9 November 2021 [16].

## 3. Clinical Overview of COVID-19

**Figure 2:**
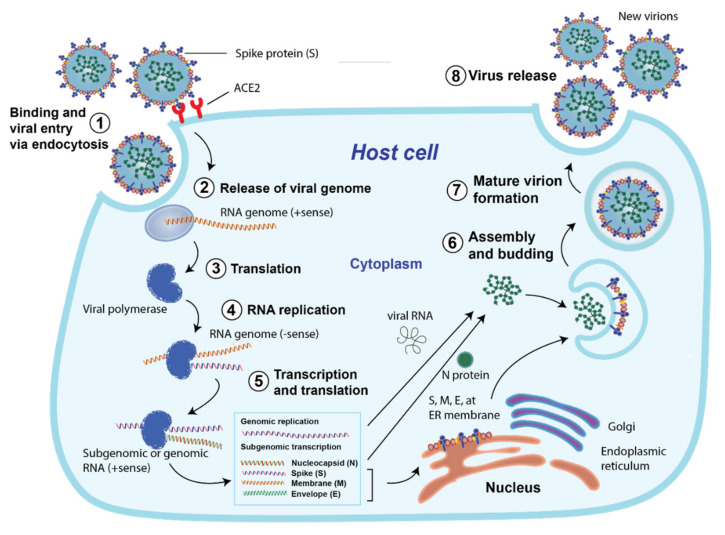
SARS CoV-2 life cycle: (1) Binding and viral entry to the host cell via endocytosis; (2) release of viral genome; (3) translation and (4) replication of RNA genome; (5) transcription and translation of RNA into protein; (6) assembly and budding, followed by (7) mature virion formation; and (8) release of the new virion from the host cell. (Redrawn by the authors based on second figure in Ref. [22]).

As with other coronaviruses, SARS-CoV-2 uses its transmembrane S protein to enter host cells (Figure 2). It binds with high affinity to human Angiotensin Converting Enzyme 2 (ACE2), ACE2 receptors, which acts as a cellular doorway for SARS-CoV-2 to fuse and enter the target cells [20]. Once this connection is established between the virus and the ACE2 receptor, an endosomal cysteine protease, Transmembrane Serine Protease 2 (TMPRSS2), is activated, and the virus enters the host cell via endocytosis [21]. After the virus enters the cell, it hijacks the cell’s protein machinery and starts making new virus particles which are released from the host cell and infect neighboring healthy cells, causing COVID-19 infection.

COVID-19 is a highly transmissible disease that represents a severe threat to public health, causing mild or moderate symptoms in most infected individuals; in ~ 20% of cases, this can be severe [23]. The virus is primarily transmitted through respiratory droplets (aerosols) from close contact between individuals [24]. The most common method used in the diagnosis of COVID-19 is real-time reverse transcriptase polymerase chain reaction (RT-PCR) of respiratory specimens, including nasal and pharyngeal swab or lung fluids [1]. Common COVID-19 symptoms include cough, shortness of breath, fatigue, fever, and loss of taste and smell, but in some cases it can progress to pneumonia, followed by a critical condition known as acute respiratory distress syndrome (ARDS), which can potentially result in multi-organ failure (MOF) and death [1,25,26,27]. Frequency of developing severe disease increases with age and comorbidities such as hypertension, obesity, and diabetes [28].

Although COVID-19 infection lasts approximately 2 weeks in most individuals and its acute effects fade by the end of the infection, its long-term effects have been demonstrated in some individuals, mainly health care workers who have had significant exposure to the virus [29]. The most common long-term effect that COVID “Long Haulers” suffer from is dyspnea, observed in ~40% of individuals [30,31]. Risk of myocardial inflammation is also substantially increased in patients who have recovered from COVID-19 [32,33]. Other residual negative effects from COVID-19 infection include increased fatigue, muscular weakness, chest pain, chronic kidney disease, neuropsychiatric disorders, cognitive disturbances and increased risk of thromboembolism [34].

Some existing small molecule drugs have been tested against COVID-19 clinically. Due to its demonstrated potent in vitro inhibitory activity against SARS-CoV, antimalarial drug, chloroquine, was proposed as an effective treatment against COVID-19 [35]. A much less toxic derivative of chloroquine, hydroxychloroquine, gained wide interest from researchers in the early days of the pandemic; however, several randomized controlled trials and meta-analyses have clearly indicated that hydroxychloroquine and chloroquine are not associated with improved recovery or reduced mortality rates [36,37]. In light of these results, The USA Food and Drug Administration (FDA) revoked the emergency use authorization for hydroxychloroquine and chloroquine on 15 June 2020 [38].

Antiviral, remdesivir, has been effective in shortening the recovery time, decreasing the mortality rate and lowering respiratory tract infections in hospitalized adults with COVID-19 [39], and it is the only currently approved small molecule drug for use by most health regulatory authorities including European Medicines Agency (EMA) and FDA against COVID-19 [40,41]. Dexamethasone, a corticosteroid, has also shown to increase recovery of patients with severe COVID-19; however, the use of corticosteroids is not recommended in people with non-severe COVID-19 because of their potentially serious adverse effects [42]. Other drugs, recommended by The National Institute of Health (NIH) COVID-19 Treatment Guidelines Panel, to be useful depending on the severity of COVID-19, are baricitinib (an anti-inflammatory biologic for rheumatoid arthritis), tocilizumab (an anti-IL-6R monoclonal immunosuppressive antibody used for autoimmune disorders) and neutralizing SARS-CoV-2 combination monoclonal antibodies, bamlanivimab/etesevimab (Eli Lilly), casirivimab/imdevimab (Regeneron Pharmaceuticals) and sotrovimab (GSK/Vir Biotech) [43] for mild to moderate cases, in particular in the early stages of infection.

Clinical trial data released in October by pharmaceutical company Merck demonstrated that molnupiravir, another antiviral, can reduce the risk of hospitalization or death by around 50% [44]. The UK Medicines and Healthcare Products Regulatory Agency (MHRA) has granted authorization for molnupiravir as the first oral antiviral in treating mild-to-moderate COVID-19 on 4 November 2021 [45].

Pfizer has recently developed an oral small molecule antiviral drug candidate, PF-07321332, with coronavirus-specific protease inhibitory activity and the drug has entered clinical trials (ClinicalTrials.gov Identifier: NCT04756531) [46,47]. Clinical trial data published in November 2021 have shown that PAXLOVID^TM^ (PF-07321332 and ritonavir combination) has been shown to reduce the risk of hospitalization or death by 89%; however, as of 29 November 2021, it has not been approved by regulatory authorities yet [48]. Even though the advances in novel drug development are promising, they will not be able to help with the fight to stop the transmission of the virus. COVID-19 has been spreading uncontrollably since the beginning of this pandemic, and the history of infectious diseases has shown that successful vaccines against COVID-19 remain the best weapon.

## 4. Traditional Vaccine Discovery

The beginning of the modern vaccination era is attributed to the English physician Edward Jenner, dating back to 1796, who is considered to have made one of the most significant breakthroughs in public health. Prior to Jenner, however, there was a technique called ‘inoculation’ or ‘engrafting’ that was practiced widely in Asia Minor and Far East [49]. Jenner was not the first scientist to determine that individuals who had contracted cowpox did not develop smallpox infection; however, he was the first to demonstrate that using cowpox pus from an infected person, another individual could be immunized, creating the first vaccine [50]. This discovery has led many researchers around the world to focus their research on vaccination, fostering advancements in the immunology field. Smallpox, one of the deadliest infections in human history, was declared eradicated in 1980 by WHO through worldwide vaccination campaigns, and many infectious diseases such as measles, polio, diphtheria and whooping cough, once seen as a significant threat to the community, are not a public health concern anymore in most parts of the world, thanks to the continuous global vaccination efforts for decades [51].

Historically, developing a safe and effective vaccine has required ~ten years, starting from early preclinical studies and followed by Phase 1–3 human clinical trials and then the arduous authorization process by national and international regulatory bodies [52]. Phase 1 involves determining the efficacy and evaluating the basic safety of the vaccine candidate in a small group (20–200) of healthy individuals [53]. Phase 2 involves a larger sample group with several hundred individuals and aims to collect additional information on safety, immunogenicity, efficacy and appropriate dosing of the vaccine candidate [53]. Phase 3 involves thousands of individuals and assesses the safety and efficacy of the vaccine candidate in larger populations, identifies real-world reactions to the vaccine candidate and determines the effectiveness by comparing vaccinated and unvaccinated groups [53]. If these results collectively demonstrate acceptable safety and efficacy, manufacturers can then submit authorization applications to different regulatory jurisdictions.

## 5. Classes of COVID-19 Vaccines

From the early days of the pandemic, it was clear to vaccine scientists/researchers, public health agencies and political leaders across the globe that the most effective way to fight the pandemic was to develop effective vaccines. Concerted global coordination and collaboration, and injection of significant financial resources from governments as well as philanthropic individuals and institutions (like the Bill & Melinda Gates Foundation and Wellcome Trust), has led to the first vaccine against COVID-19 being approved for emergency use by MHRA on 2 December 2020 and by FDA on 11 December 2020, less than a year after the pandemic began [54,55]. As of 15 November 2021, 23 vaccines have been authorized or approved for emergency use in at least one country, most receiving Emergency Use Authorization (Table 2 and Appendix A), 122 vaccine candidates are in different phases (1–3) of clinical trials (Appendix B) and 194 vaccine candidates are in pre-clinical development phase [56,57].

**Figure 3:**
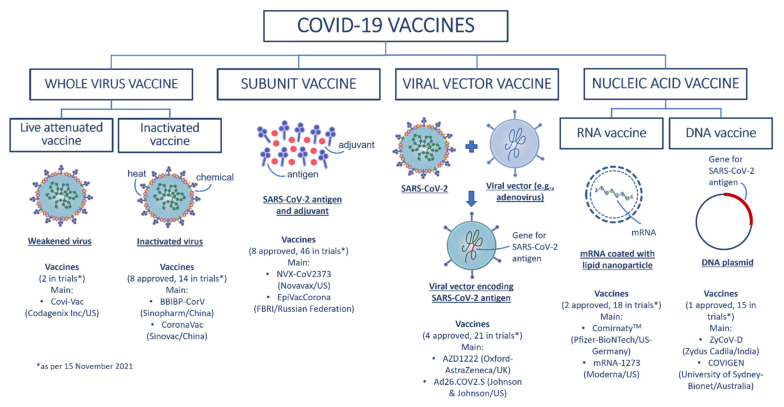
Schematic summary of the four broad groups of COVID-19 vaccines with examples of current COVID-19 vaccines. (Redrawn by the authors based on second figure in Ref. [60]).

COVID-19 vaccines and vaccine candidates may be categorized into four groups based on their development technology. In addition to traditional vaccine development platforms such as inactivated and live attenuated vaccines, novel methods including nucleic acid vaccines and viral vector vaccines are also being used in the development of COVID-19 vaccines. The four broad groups of COVID-19 vaccines are summarized in Figure 3 followed by a brief description of these vaccine types.

### 5.1. Whole Virus COVID-19 Vaccines

#### 5.1.1. Inactivated Vaccines

Inactivated vaccines are produced by using heat, radiation or chemicals, such as formaldehyde or β-propiolactone, to break down the viral structure and/or genetic material [58]. These vaccines still contain all parts of the virus, but in an inactive form making the virus unable to cause human disease. Inactivated vaccines are generally considered safe, easy to develop and manufacture and are less immunogenic; as such, they may not induce a strong enough immune response, which would necessitate addition of adjuvants and/or multiple doses [59].

Since the start of the COVID-19 pandemic, inactivated vaccine development has been the preferred vaccine development platform by some companies since it is the most established methodology with easy development and manufacturing of vaccines without compromising safety. As of 15 November 2021, there are 17 inactivated COVID-19 vaccines and/or vaccine candidates being tested in different phases of clinical trials. 8 of these 16 vaccines; Sinopharm-BBIBP-CorV (Sinopharm, Beijing, China), CoronaVac (Sinovac, Beijing, China), Covaxin (Bharat Biotech, Hyderabad, India), Sinopharm-WIBP (Sinopharm, Beijing, China), CoviVac (Chumakov Center, Moscow, Russia), QazVac (Research Institute for Biological Safety Problems, Zhambyl, Kazakhstan), COVIran Barakat (Shifa Pharmed Industrial Co, Kordan, Iran) and another vaccine developed by Shenzhen Kangtai Biological Products, China have been granted emergency use authorization in at least one country [57]. However, only three of these vaccines, Sinopharm-BBIBP-CorV, Sinopharm-WIBP, and CoronaVac, were included in the WHO Emergency Use Listing (EUL) released on 12 November 2021 [61].

Clinical trials of CoronaVac were conducted in more than 10 million individuals from four countries: Chile, Indonesia, Brazil and Turkey. Results from these trials show that the effectiveness of vaccination varies between 50% and 84%; however, the efficacy of vaccine against hospitalization was superior, 85% in Chile and 100% in Brazil and Turkey. Sinopharm-BBIBP-CorV was found to be 78% effective in phase 3 trials that were conducted in approximately 40,000 participants from the United Arab Emirates and Bahrain [62].

According to a survey of 1526 individuals who had CoronaVac, the most common side effect after the vaccination was localized pain at the injection site, which accounted for ~70% of the total reported side effects, that was experienced by 15% of the vaccinated group. Other notable side effects were fatigue, muscle pain and dizziness, which were experienced by 8.3%, 8.1% and 6% of the group, respectively [63]. Of the 35.8 million doses that have been administered since CoronaVac authorization in China by March 2021, the number of severe adverse effects reported was only 49 [64].

#### 5.1.2. Live Attenuated Vaccines

Live attenuated virus vaccines are developed by attenuating the viruses, usually by repeated culturing. These vaccines generate a strong immune response and in most attenuated vaccines immunity is produced with a single dose. This strong immune response, however, can cause unwanted effects resulting in limited use of these vaccines in individuals with compromised immune status. This vaccine development method is the least preferred of the existing methods and as of 15 November 2021, only two COVID-19 vaccine candidates, which are in phase 3 and 1 trials, were developed using this method [65].

### 5.2. Subunit Vaccines

Unlike traditional platforms, a subunit vaccine only uses a part of the virus, as antigen, to stimulate immune response. This type of vaccine is developed using recombinant proteins or synthetic peptides targeting specific epitope, therefore eliminating the potential risk of pathogenicity and thus minimizing side-effects [65]. Despite such advantages, the ability of subunit vaccines to trigger immune response are lower than those vaccines that contain the entire virus. Thus, multiple doses and adjuvant(s) are often needed [66,67]. Several types of COVID-19 subunit vaccines are being developed: protein subunit vaccines containing specific isolated proteins from virus; polysaccharide vaccines that contain chains of sugar molecules; conjugate subunit vaccines in which a polysaccharide chain is attached to a carrier protein; and virus-like particles (VLPs) which mimic the structure of actual virus particles [68].

The S protein is used as the pivotal target for developing subunit vaccines against SARS-CoV-2, as it plays a key role in receptor binding and serves as the major antigen that triggers the response of protective neutralizing antibodies [69]. As of 15 November 2021, eight protein subunit vaccines against SARS-CoV-2 have been approved for emergency use at least in one country, such as RBD-Dimer, developed by Anhui Zhifei Longcom Biopharmaceutical, that uses a dimeric form of the S protein RBD. EpiVacCorona, developed by Vektor State Research Center of Virology and Biotechnology in Russia, utilizes a synthetic peptide antigen of SARS-CoV-2 containing a carrier protein and an adjuvant (Table 2).

### 5.3. Viral Vector Vaccines

Viral vectors are viruses that have been genetically engineered to create vaccines. They are completely harmless and are used as carriers to deliver the genetic information which the host cell uses to produce the antigen that initiates the body’s immune response [70]. The concept of using a virus as a vector dates back to 1972 [71]; however, it was not until 2019 that the first viral vector vaccine, ERVEBO^®^ vaccine against Ebola virus, was approved for human use [72]. Several viruses such as Retrovirus, Lentivirus, Cytomegalovirus and Adenovirus have been used as carriers. Adenovirus is the most frequently used viral vector due to its well-established safety profile and inflammatory and immune system triggering effects [70].

This vaccine development platform is one of the most common used technologies for the development of COVID-19 vaccines; a number of vaccines developed with this approach are approved for emergency use in several countries and this method will remain a promising vaccine development pathway for ongoing vaccine research [57]. As of 15 November 2021, 21 viral vector vaccines have entered clinical trials and four of these vaccines; ChAdOx1-S (Oxford-AstraZeneca, Cambridge, UK and Stockholm, Sweden), Sputnik V (Gamaleya Research Institute, Moscow, Russia), Ad26.COV2.S (Johnson & Johnson, New Brunswick, NJ, USA) and Convidecia (CanSino Biologics, Shanghai, China) have been approved for either emergency or full use in different countries [65].

ChAdOx1-S by Oxford-AstraZeneca was approved for emergency use against COVID-19 on 30 December 2020 and in the subsequent 6 months it has become the most widely approved COVID-19 vaccine internationally [57]. Clinical trials have demonstrated that the vaccine is 66% effective after the second dose against COVID-19 infection with no hospital admissions in the vaccinated group [73]. However, it has been associated with venous thromboembolism, coagulation disorders and blood clots. According to a study that examined 280,000 vaccinated individuals, 59 venous thromboembolic events were observed in the vaccinated cohort compared with 30 expected based on the incidence rates in the general population, corresponding to a standardised morbidity ratio of 1.97. The same study also demonstrated that the standardised morbidity ratio for any thrombocytopenia/coagulation disorders was 1.52, indicating an increased risk of around 50% [74]. In some cases, these adverse events have led to several deaths, which has prompted nine countries in Europe to suspend emergency use approval of this vaccine [75]. A recent study demonstrated that ChAdOx1 viral vaccine vector binds to platelet factor 4 (PF4), a protein involved in the pathogenesis of heparin-induced thrombocytopenia (HIT), which could be a major step in discovering the mechanism underlying this rare side effect [76].

### 5.4. Nucleic Acid COVID-19 Vaccines

Nucleic acid-based vaccines have received much interest in the field of new vaccines, following studies in the early 1990s that plasmid DNA induces an immune response to the plasmid-encoded antigen and a mRNA vaccine was found to be effective as a result of direct gene transfer [47,77,78].

Nucleic acid vaccines use a part of genetic material in the form of DNA (as plasmids) or RNA (as mRNA) which encode and are translated in cells to a specific protein to stimulate immune responses. They have significant benefits over conventional methods in terms of safety (live virus and adjuvant are not required), effectiveness (expressing antigen in situ and mimicking true infection, thus inducing both B and T-cell responses), and high specificity (inducing immune response to the antigen of interest only) [79]. Additionally, it is relatively cheap and requires a shorter time to develop and manufacture this type of vaccine compared to traditional vaccines.

#### 5.4.1. DNA Vaccines

DNA vaccines are formulated in the form of plasmids expressing the specific target protein (antigen). This process requires the intermediary steps of translation of DNA into messenger RNA which carries the specific genetic information (code) to the ribosomes where the protein synthesis takes place [80]. As per 15 November 2021, a DNA vaccine candidate against SARS-CoV-2, ZyCoV-D, has been approved for emergency use in India (Table 2) and several others are being developed and are entering clinical trials (Appendix B), like AG0301-COVID19 (Phase 2/3—AnGes, Tottori, Japan), and Covigenix VAX-001 (Phase 1—Entos Pharmaceuticals Inc, Edmonton, AB, Canada).

#### 5.4.2. RNA Vaccines

RNA vaccine in the form of mRNA involves an intermediate process between DNA and protein translation by ribosomes. Currently, there are two main types of mRNA vaccines being studied: non-replicating mRNA, and self-amplifying RNA. The conventional non-replicating mRNA containing 5′ and 3′ untranslated region (UTRs) work by encoding the protein of interest, whereas the self-amplifying RNA encodes not only the protein/antigen, but also the viral replication machinery enabling the intracellular RNA amplification and large amounts of protein expression [81].

Although they work in similar manner, RNA-based vaccines appear to be more effective and safer than DNA vaccines; injection of RNA poses no (or minimal) risk of disrupting original DNA sequences in cells since it does not need to enter the cell nucleus [82]. The mRNA vaccines have become one of the leading vaccines to be developed against SARS-CoV-2. Two mRNA vaccines: BNT162b2 (Pfizer/BioNTech, New York, NY, USA/Berlin, Germany) and mRNA-1273 (Moderna, Cambridge, MA, USA) have been approved for emergency use worldwide (Table 2). Recently, BNT162b2 (Pfizer/BioNTech, New York, NY, USA/Berlin, Germany) vaccine received full approval from the FDA.

**Table 2:** Authorized/approved vaccines in at least one country as per 15 November 2021.

No	Vaccine Name	Status	Developer	Vaccine Type	Efficacy	Dose	Storage	Price (per Dose)	Source
1	Comirnaty^TM^ (BNT162b2)	Approved in several countries, emergency use in US, elsewhere	Pfizer-BioNTech Germany-US	RNA based vaccine	95% [83]	2 dose, 3 weeks apart [84]	−70 °C [85]	€19.50 (US$23.15) [86]	[56]
2	Moderna mRNA-1273 and mRNA-1273.351	Approved in Switzerland, emergency use in US, elsewhere	ModernaTX, Inc US	RNA based vaccine	94.1% [87]	2 doses, 4 weeks apart [88]	−25 °C [89]	US$25.50 [86]	[56]
3	AstraZeneca AZD1222	Approved in Brazil, emergency use in EU, elsewhere	The University of Oxford-AstraZeneca UK	Viral vector (non-replicating)	76% [90]	2 doses, between four and 12 weeks apart [91]	2–8 °C [85]	US$2.15 (EU), US$5.25 (others) [92]	[56]
4	Convidecia^TM^ (Ad5-nCoV)	Approved in China, emergency use in other countries	CanSino Biologics China	Viral vector (non-replicating)	65.28%	Single dose	2–8 °C [93]	US$27.15 (Pakistan) [94]	[56]
5	Ad26.COV2.S	Emergency use in US, elsewhere	Janssen (Johnson & Johnson) US	Viral vector (non-replicating)	66.9% [95]	Single dose [95]	2–8 °C [85]	US$10 [96]	[56]
6	BBIBP-CorV	Approved in China, Bahrain, UAE, emergency use in other countries	Sinopharm (Beijing) China	Inactivated virus	79% [97]	2 doses, 3 weeks apart [98]	2–8 °C [99]	US$37.50 (Hungary) [100]	[56]
7	Inactivated SARS-CoV-2 (vero cell)	Approved in China, Limited use in UAE	Sinopharn + Wuhan Institute of Biological Products China	Inactivated virus	72.8%	2 doses, 3 weeks apart	2–8 °C	N/A	[56]
8	CoronaVac	Approved in China, emergency use in other countries	Sinovac China	Inactivated virus	51% in Brazil trial, 84% in Turkey trial [99]	2 doses, 2 weeks apart [99]	2–8 °C [89]	US$13.60 (Indonesia) [101]	[56]
9	Sputnik V	Emergency use in Russia, elsewhere	The Gamaleya Research Institute Russia	Viral vector (non-replicating)	91.6%	2 doses, 3 weeks apart	−18 °C [89]	Less than US$10 [102]	[103]
10	EpiVacCorona	Approved in Turkmenistan, early use in Russia	FBRI Russia	Protein subunit	N/A	2 doses, 3 weeks apart	2–8 °C	US$11 [104]	[103]
11	ZF2001/RBD-Dimer	Emergency use in China, Uzbekistan	Anhui Zhifei Longcom China	Protein subunit	N/A	3 doses, 4 weeks apart	2–8 °C	N/A	[103]
12	Soberana 2/Pasteur	Emergency use in Iran, Cuba	Instituto Finlay de Vacunas Cuba	Protein subunit	62% two doses, 91.2% with Soberana Plus	2 doses, 4 weeks apart	2–8 °C	N/A	[103]
13	Abdala/CIGB-66	Emergency use in Cuba	Center for Genetic Engineering and Biotechnology (CIGB) Cuba	Protein subunit	92.28%	3 doses, 2 weeks apart	2–8 °C	N/A	[103]
14	Medigen	Emergency use in Taiwan	Medigen Vaccine Biologics Taiwan	Protein subunit	N/A	2 doses, 4 weeks apart	2–8 °C	N/A	[56]
15	Covaxin^®^	Emergency use in India, elsewhere	Bharat Biotech India	Inactivated virus	77.8%	2 doses, 4 weeks apart	At least a week at room temperature	US$16.42 (India) [105]	[103]
16	QazCovid-in^®^	Early use in Kazakhstan	Research Institute for Biological Safety Problems Kazakhstan	Inactivated virus	N/A	1 or 2 doses, 3 weeks apart	2–8 °C	US$4.7 [106]	[103]
17	Inactivated (Vero Cells)	Emergency use in China	Shenzhen Kangtai Biological Products Co., Ltd. China	Inactivated virus	N/A	2 doses, 4 weeks apart	2–8 °C	N/A	[103]
18	COVIran Barekat	Emergency use in Iran	Shifa Pharmed ParsIran	Inactivated virus	N/A	2 doses	2–8 °C	N/A	[103]
19	CoviVac	Early use in Russia	Chumakov Cente Russia	Inactivated virus	N/A	N/A	2–8 °C	N/A	[103]
20	NVX-CoV2373	Emergency use in Indonesia	Novavax US	Protein subunit	89.7%	2 doses, 3 weeks apart	2–8 °C	US$20.90 (Denmark) [86]	[103]
21	ZyCoV-D	Emergency use in India	Zydus CadilaIndia	DNA based vaccine	66.6%	3 doses, 4 weeks apart	2–8 °C	N/A	[103]
22	COVAX-19^®^	Emergency use in Iran	Vaxine Pty.Ltd/Cinnagen Co. Australia	Protein Subunit	N/A	2 doses, 3 weeks apart	N/A	N/A	[103]
23	Soberana Plus	Emergency use in Cuba	Instituto Finlay de Vacunas Cuba	Protein subunit	N/A	N/A	N/A	N/A	[103]

## 6. COVID-19 Vaccines—Future Outlook

### 6.1. Combination COVID-19 Vaccines

When faced with evolving safety issues and variable supply and logistical challenges with current COVID-19 vaccines, combination COVID-19 vaccinations have emerged as superior alternatives for providing individuals with the immune protection required. A research study carried out in March 2021 revealed that combining several distinct COVID-19 vaccines increased immune responses in mice [107]. Consecutive immunization with an adenovirus vector vaccine followed by inactivated/recombinant subunit/mRNA vaccine particularly enhanced neutralizing antibody levels and promoted antibody response to primary neutralizing antibodies. Preliminary results of the CombiVacS trial, which included over 600 patients in Spain, are the first to demonstrate the benefits of mixing different coronavirus vaccines [108]. Other studies also support this concept in that combining the Oxford-AstraZeneca vaccine and the Pfizer-BioNTech vaccine induces a stronger immune response compared to two doses of the same vaccine [109,110,111]. However, while no serious side effects have been recorded in mix-and-match trials, some safety concerns remain when combining two distinct vaccines, as each vaccine has its own set of adverse events/side effects.

### 6.2. Booster COVID-19 Vaccines

SARS-CoV-2 B.1.617.2 variant, also known as the Delta variant, was first detected in India in late 2020; quickly spreading to many countries and becoming the dominant variant by mid-2021. Due to its higher transmissibility, this new strain caused a resurgence in the number of COVID-19 cases, particularly in regions with lower vaccination rates [112,113]. Although existing vaccines like BNT162b2 and ChAdOx1 are effective against this new Delta variant, 88% and 67% respectively, the efficacy of BNT162B2 was reduced from 94% to 88% and from 74% to 67% for ChAdOx1 compared to their respective effectiveness against the earlier alpha variant [114]. Breakthrough infections caused by the Delta variant among vaccinated healthcare workers have also been reported in several countries [115,116]. The resurgence in COVID-19 cases and the possibility of reductions in the protective effects of the vaccines over time [117], combined with the Delta variant’s higher transmissibility and increased risk of infection among vaccinated individuals have prompted health authorities to consider introducing booster vaccines.

Recent studies demonstrated that BNT162b2 booster shot significantly reduces the risk of COVID-19 infection and severe illness [118]. Another study tested seven different vaccines (BNT162b2, ChAdOx1-S, mRNA-1273, NVX-CoV2373, Ad26.COV2.S, CVnCoV and VLA2001) as boosters and showed that all seven of them boosted the immunity in both older and younger populations [119]. A number of countries, such as Israel, UAE, Russia and Turkey, had already commenced administering booster shots to high-risk individuals and healthcare professionals, even before the data were published, with an effort to counteract the decrease in immunity over time by standard 2-dose vaccination [120,121,122]. FDA released a statement on 12 August 2021, authorizing booster vaccines for immunocompromised individuals [123]. Both FDA and EMA have recently approved the use of BNT126b2 and mRNA-1273 as booster vaccines, and as of November 2021, many countries from all the continents around the world are offering their citizens booster shots [120]. WHO on the other hand, is questioning the urgency for booster doses with concerns that such practice would further increase disparities in vaccination rates in many low income countries, and preventing the ultimate international public health goal of high vaccination rates globally [124].

### 6.3. Mandatory COVID-19 Vaccinations

Another strategy being implemented by several countries and organizations to reduce the spread of COVID-19 is the mandatory vaccination of individuals working in certain sectors such as healthcare, transportation, education and retail. Tajikistan has become the first country to make the COVID-19 vaccines mandatory for all its citizens over 18 years of age [125]. Turkmenistan has been reluctant to share data on COVID-19 cases and deaths since the first days of the pandemic; however, it became the second country to make COVID-19 vaccines mandatory for all its citizens over 18 years of age [126]. Saudi Arabia has imposed mandatory vaccination for all individuals wanting to enter any government or private facility and made full vaccination mandatory for participation in Hajj [127]. Italy, France, Greece and the UK are making it mandatory for certain healthcare workers to be fully vaccinated and are imposing vaccination requirements for many social gatherings and venues including cinemas, bars, clubs and other closed spaces [128]. In the United States, California and New York are the only two states so far to impose a vaccine mandate, requiring all government officials to get fully vaccinated [129]. While these measures have resulted in noticeable increases in vaccination rates, they have also sparked protests around the world, mostly by anti-vaccination activists [130].

## 7. COVID-19 Vaccination Consideration

### 7.1. Uniform Vaccine Availability and Affordability

The development, testing and approval of several vaccines in the space of 18 months is an extraordinary scientific achievement and will be remembered as a milestone in the history of pandemics. Within 8 months, nearly 5 billion doses of vaccines have been administered since the authorization of the first vaccine [3]. Although this number is impressive for such a short time-period, it is not an accurate indicator of the global availability of vaccines. The increased movement of individuals globally, the economic and social interdependencies between countries and the ease of travel will make it even harder to contain the spread of the virus unless the global immunity goal is achieved. To reach the global immunization goal, a huge stepwise increase in the number of vaccines is required, in every region, state and country, particularly in areas with low vaccination rates.

COVID-19 Vaccines Global Access (COVAX) is a worldwide initiative co-led by Gavi the Vaccine Alliance, Coalition for Epidemic Preparedness Innovations (CEPI) and WHO, with UNICEF as the delivery partner; COVAX aims to accelerate the development, manufacturing of COVID-19 vaccines and ensure equitable access for every country in the world [131]. High Income Countries such as UAE, Qatar and Israel have been able to vaccinate more than 70% of their population; however, this target declines to below 1% in Low- and Middle-Income Countries such as DR Congo, South Sudan and Yemen [3]. One of the goals of COVAX is to ensure that at least 20% of each country’s population receives vaccines. As of 24 August 2021, COVAX has delivered 215 million COVID-19 vaccines to nearly 100 countries with the aim of providing 1.9 billion doses by the end of 2021 [132,133].

The currently available COVID-19 vaccines cost in the order of US$2 to US$37 per dose (Table 2). Many western countries (like the US, UK and Australia) either offer these vaccines free of charge to their citizens or subsidize such vaccination programs for the public good. Government or third-party payer subsidized vaccination schemes are designed to absorb the cost of COVID-19 vaccines and to encourage greater participation in COVID-19 vaccinations. According to WHO’s report released in April 2021, more than 87% of the of the vaccine doses have gone to either high income or upper-middle income countries [134]. This issue needs to be addressed. From an ethical perspective, regardless of their wealth or economic situation every individual’s access to appropriate basic health care needs to be guaranteed, especially from the perspective of infectious diseases during a global pandemic, since at least a certain percentage of each population needs to be vaccinated to confer herd immunity and to combat a pandemic. In addition, western and other high-income countries have much higher drug budgets and are also able to move funds quickly to COVID-19-related activities (testing, vaccine administration programs and/or providing/expanding ICU facilities) as the pandemic evolves. This is neither possible or feasible in many low- and middle- income countries where unfortunately drug/health budgets are smaller and government subsidies or third-party insurers do not operate in the health market. It is therefore critical for world agencies (like WHO, COVAX and the UN) to find equitable solutions to not only timely availability of COVID-19 vaccines for all countries but also at an affordable price so that herd immunity to COVID-19 is reached in all countries. This is especially critical as new variants of the virus emerge which will require additional vaccines beyond the initial vaccination cycle.

### 7.2. Vaccine Hesitancy

The Strategic Advisory Group of Experts on Immunization (SAGE) is a working group whose mission is to advise WHO on global policies and strategies concerning all vaccine-preventable diseases and SAGE defines vaccine hesitancy as “*delay in acceptance or refusal of vaccination despite availability of vaccination services*” [135]. SAGE was established in 1999; however, emergence of vaccine hesitancy and anti-vaccine (anti-vax) movement(s) can be traced to Jenner’s time (18th century), with respect to early smallpox vaccination programs. Factors including religious bigotry (injecting a purulent matter from a lower species to humans), substandard methods used in vaccination and the 1853 Compulsory Vaccination Act being seen as an example of class legislation-initiated public mistrust against vaccination have all given rise to anti-vaccine campaigns [136].

Reluctance to vaccination, in particular essential childhood vaccines like measles, has been rising in recent years [137]. Genuine public discourse about vaccine efficacy and side effects and other safety issues has translated to rising levels of global vaccine hesitancy. Safety of vaccines and vaccination programs have become paramount public health issues that need to be addressed urgently. Major public concerns have arisen for political, religious, socio-economic or philosophic reasons and include, for example, questions about use of cell cultures from human or animal embryo, which are used commonly to grow the virus for vaccine preparation [138,139]. It is worth emphasizing that vaccines undergo rigorous safety and efficacy evaluation and their benefits far outweigh their risks as part of mass vaccination programs. Some infections like measles require high levels of herd immunity to prevent community spread. Achieving such herd immunity is a prerequisite to protecting immune-compromised individuals or others who cannot be vaccinated. Inadequate herd immunity is probably the major reason why recent measle outbreaks have been observed in some tight-knit communities in the US, such as Ultra-orthodox Jewish populations in New York and other US states [140].

The anti-vaccine movement gained significant momentum in the 1990s due mainly to publications that appeared in Lancet that was retracted ~12 years later. Andrew Wakefield, a UK physician reported that measles, mumps and rubella (MMR) vaccine caused autism in eight children [141]. Even though this was hypothesized in a small number of children, Wakefield’s claims attracted widespread interest, first in the UK but then globally, and resulted in parents either refusing or delaying MMR vaccines and sometimes even other vaccines as well. An upsurge in measles in the UK, EU and particularly in the USA coincided with Wakefield’s erroneous claims. Large-scale studies subsequently refuted Wakefield’s claims and proved that vaccines were not causing autism [142].

With the proliferation of social media and its rapid and immediate influence, and with the involvement of public figures (e.g., prominent politicians and Hollywood actors), the anti-vax movement has gained an even stronger hold in some regions of the world and has garnered new followers or at least empowered many to be highly skeptical about vaccine safety [143,144,145]. Currently, there are many online anti-vax campaigns, podcasts, blogs, videos, articles and books [146]. Some “anti-vaxxers” are highly ‘educated’ in non-medical topics and claim that they would like to protect their children from vaccine side-effects. It is unfortunate, from a public health perspective, that many individuals remain skeptical about the effectiveness and safety of vaccines. A more sophisticated education and behavior-change approach is necessary to educate the public about overall vaccine development, how vaccines work, nature of vaccine clinical trials and their public health utility as well as their shortcomings, so individuals can base their decisions regarding vaccination on objective scientific evidence and clear public health messages. The potential harmful effects of vaccine hesitancy on global public health has become so evident that the WHO has listed vaccine hesitancy as one of the top ten threats to global health in 2019 [147].

As a result, immunization of many politicians and other prominent public figures with COVID-19 vaccines has been highly publicized to highlight their overall safety and to ensure greater COVID-19 vaccine uptake. Based on surveys that were conducted in 2020, the acceptance rates of COVID-19 vaccines differ dramatically across countries around the world with the highest rate seen in Ecuador (97%) [148] and the lowest rate in Kuwait (23%) [149]. In a study in healthcare workers, the highest COVID-19 vaccine acceptance rate (94%) was in those working in COVID-19 departments compared to 77% in healthcare professionals working in non-COVID-19 departments [150]. A systematic review [151] has shown that the population intending to get a vaccine has declined as the pandemic has progressed, which is likely to be attributed to both increased exposure to misinformation and concerns over the safety of the vaccines [152,153]. The same study demonstrated that generally being female, younger, of lower income or education status or belonging to an ethnic minority group were associated with increased vaccine reluctance. A systematic review of COVID-19 vaccine hesitancy in the US population has shown a similar result with the highest hesitancy rate being in Black/African Americans and pregnant or breastfeeding women [154]. In the case of COVID-19 vaccines, public health authorities not only have to deal with existing prejudices of anti-vaxxers such as political, religious or cultural beliefs, they also need to convince the public that, with advanced vaccine development technologies and the substantial funding that have been provided to the scientific community, it is now possible to develop safe and effective COVID-19 vaccines in much shorter timeframes without compromising effectiveness and more importantly safety.

## 8. Conclusions

Despite stepwise technological advancements in public health and medicine, substantial increases in number of well-trained health personnel, facilities and sophisticated medical equipment and increased access to and sharing of up-to-date scientific and medical information in many countries, the COVID-19 pandemic has proven that pandemic preparedness is still a major global issue that needs to be addressed urgently. SARS, MERS, Ebola, and various influenza outbreaks were able to be contained before evolving into global pandemics; however, it is clear from the ongoing COVID-19 pandemic that the world needs to revisit its emergency plans and improve its preparedness for potential similar future outbreaks.

One striking shining light during this pandemic has been the timely development and commercial availability of COVID-19 vaccines. This has largely been possible due to targeted international collaborative research and scientific efforts and availability of significant financial resources from governments, individuals, institutions/international organizations and philanthropists. Although developing COVID-19 vaccines in less than a year has been one of the significant breakthroughs in the history of medical science, with the emergence of new COVID-19 variants, future research needs to specifically focus on vaccines that have the potential to act against new variants to either stop transmission or infection.

Nevertheless, many major issues remain, including uneven availability/distribution of such vaccines across the globe, their affordability and the increase in vaccine hesitancy. These issues will require strong political will and leadership and concerted public health initiatives and messaging if the full potential of COVID-19 vaccines is to be realised for all individuals in all countries irrespective of socio-economic status and cultural and/or religious backgrounds. This is the challenge that lies ahead.

## Figures and Tables

**Table 1:** SARS-CoV-2 Variants Being Monitored (VBM) and Variants of Concern (VOC) [15,17,18].

	WHO Terminology	Pangolin *	S Protein Mutations of Interest	Country of First Detection	Time of First Detection
VBM	Epsilon	B.1.427/B.1.429	L452R, D614G, S13I, W152C, L452R, D614G	United States (California)	September 2020
	Eta	B.1.525	A67V, 69del, 70del, 144del, E484K, D614G, Q677H, F888L	United Kingdom/Nigeria	December 2020
	Iota	B.1.526	L5F, (D80G*), T95I, (Y144-*), (F157S*), D253G, (L452R*), (S477N*), E484K, D614G, A701V, (T859N*), (D950H*), (Q957R*)	United States (New York)	November 2020
	Kappa	B.1.617.1	(T95I), G142D, E154K, L452R, E484Q, D614G, P681R, Q1071H	India	December 2020
	Zeta	P.2	E484K, (F565L*), D614G, V1176F	Brazil	April 2020
	Lambda	C.37	G75V, T76I, 246-252del, L452Q, F490S, D614G and T859N	Peru	December 2020
	Mu	B.1.621, B.1.621.1	N/A	Colombia	September 2021
	Alpha	B.1.1.7	69del, 70del, 144del, (E484K*), (S494P*), N501Y, A570D, D614G, P681H, T716I, S982A, D1118H (K1191N*)	United Kingdom	September 2020
	Beta	B.1.351	D80A, D215G, 241del, 242del, 243del, K417N, E484K, N501Y, D614G, A701V	South Africa	May 2020
	Gamma	P.1	L18F, T20N, P26S, D138Y, R190S, K417T, E484K, N501Y, D614G, H655Y, T1027I	Japan/Brazil	November 2020
VOC	Delta	B.1.617.2	T19R, (V70F*), T95I, G142D, E156-, F157-, R158G, (A222V*), (W258L*), (K417N*), L452R, T478K, D614G, P681R, D950N	India	October 2020
	Omicron	B.1.1.529	N/A	South Africa	November 2021

* The genetic lineages of SARS-CoV-2 variants are classified using a software tool named PANGOLIN (Phylogenetic Assignment of Named Global Outbreak Lineages) developed by Andrew Rambaut’s laboratory in the United Kingdom. This classification system has since been used by researchers and public health agencies worldwide to monitor the transmission and spread of SARS-CoV-2 [19].

## Data Availability

Not applicable.

## Appendix A

**Table A1:** More information on authorized/approved vaccines in at least one country as per 15 November 2021.

No	Vaccine Name	Age Group	Common Side Effect(s)	Main User–Country
1	Comirnaty^TM^ (BNT162b2)	5 years of age and older [84]	Pain at the injection site, tiredness, headache, muscle pain, chills, joint pain, and fever	US, UK, South Korea, Singapore, Saudi Arabia, New Zealand, Japan, Israel, Hungary, Germany, France, Canada, Australia
2	Moderna COVID-19 VaccinemRNA-1273 and mRNA-1273.351	18 years of age and older [88]	Pain at the injection site, tiredness, headache, muscle pain, chills, joint pain, swollen lymph nodes in the same arm as the injection, nausea and vomiting, and fever	US, UK, Singapore, France
3	COVID-19 Vaccine AstraZeneca (AZD1222)	18 years of age and older [91]	Injection site pain or tenderness, tiredness, headachemuscle pain, fever and chills	UK, South Korea, Saudi Arabia, Phillipines, India, Germany, France, Canada, Brazil, Australia
4	Convidecia^TM^ (Ad5-nCoV)	18 years of age and older [155]	Injection site pain, mild to severe fever (up to grade 3), headache, mild to severe fatigue (up to grade 3), muscle and joint pain, throat pain and cough.	China, Pakistan
5	Ad26.COV2.S	18 years of age and older [95]	Arm (pain, redness, swelling), body (tiredness, headache, muscle pain, chills, fever, nausea)	US, South Africa, The Netherlands
6	BBIBP-CorV	18 years of age and older [98]	Headaches, fatigue, injection site reactions	China, Hungary, UAE
7	Inactivated SARS-CoV-2 (vero cell)	18 years of age and older	Injection site pain, followed by fever, which were mild and self-limiting	China, UAE
8	CoronaVac	18 years of age and older [99]	Injection site reactions, fatigue, diarrhea, and muscle pain	China, Brazil, Turkey, Indonesia, Phillipines
9	Sputnik V	N/A	Headaches, pain at injection site	Russia
10	EpiVacCorona	N/A	N/A	Turkmenistan, Russia
11	ZF2001/RBD-Dimer	N/A	Common mild side-effects including injection pain, redness and swelling	China, Uzbekistan
12	Soberana 2/Pasteur	N/A	Pain and redness at the injection site, general malaise	Iran
13	Abdala/CIGB-66	N/A	No serious adverse side effect	Cuba
14	Medigen	N/A	No vaccine-related serious adverse effects	Taiwan
15	Covaxin^®^	18 years of age and old-er [156]	Fever, headaches, irritability, pain, swelling, or both at the site of injection	India
16	QazCovid-in^®^	N/A	No serious side effects	Kazakhstan
17	Inactivated (Vero Cells)	N/A	N/A	China
18	COVIran Barekat	N/A	N/A	Iran
19	CoviVac	N/A	N/A	Russia
20	NVX-CoV2373	N/A	N/A	Indonesia, Denmark
21	ZyCoV-D	N/A	N/A	India
22	COVAX-19^®^	N/A	N/A	Iran
23	Soberana Plus	N/A	N/A	Cuba

## Appendix B

**Table A2:** COVID-19 vaccine candidates in phase 1–3 clinical trial as per 15 November 2021 [56].

No	Vaccine Candidate	Developer	Vaccine Type	Developer Country	Trial Phase	Clinical Trial ID	Country	Enrolment Target	Actual/Estimated Trial Dates	Primary Outcome Measures
1	Gam-COVID-Vac Sputnik V	Gamaleya Research Institute	Viral vector (non-replicating)	Russia	3	NCT04530396	Russia	33,758	7 September 2020–1 May 2021	Percentage of trial subjects with COVID-19 developed within 6 months after the first dose
2	NVX-CoV2373	Novavax	Protein subunit	US	3	NCT04611802	Mexico, Puerto Rico, US	33,000	27 December 2020–30 June 2023	Participants with symptoms; reactogenicity incidence and severity; incidence and severity of MAAEs, UnSoAEs, SAEs, AESIs; antibodies to SARS-CoV-2 Nucleoprotein (NP); deaths due to any cause
3	ZF2001/RBD-Dimer	Anhui Zhifei Longcom	Protein subunit	China	3	NCT04646590	China, Ecuador, Indonesia, Pakistan, Uzbekistan	29,000	16 December 2020–April 2022	Endpoints of efficacy and safety
4	CVnCoV	Curevac	RNA based vaccine	Germany	3	NCT04652102	Germany	36,500	14 December 2020–15 May 2022	Participants with virologically confirmed PCR positive cases of COVID-19 of any severity; participant with MAAEs, SAEs, AESIs
5	Inactivated (Vero Cells)	Chinese Academy of Medical Sciences	Inactivated virus	China	3	NCT04659239	Brazil, Malaysia	34,020	28 January 2021–July 2022	Incidence of COVID-19 cases after two-doses of vaccination, the incidence of SoAEs
6	QazCovid-in^®^	Research Institute for Biological Safety Problems Kazakhstan	Inactivated virus	Kazakhstan	3	NCT04691908	Kazakhstan	3000	25 December 2020–30 July 2021	Seroconversion; vaccine immunogenicity versus placebo, frequency of confirmed COVID-19 cases
7	ZyCoV-D	Zydus Cadila	DNA based vaccine	India	3	CTRI/2021/01/030416	India	28,216	N/A	To demonstrate the efficacy of ZyCoV-D in the prevention of virologically confirmed symptomatic COVID-19 cases as compared to placebo
8	Covaxin	Bharat Biotech	Inactivated virus	India	3	NCT04641481	India	25,800	16 November 2020–December 2022	First occurrence of virologically confirmed (RT-PCR positive) symptomatic COVID-19 cases
9	VAT00002: with adjuvant	Sanofi/GSK	Protein subunit	US	3	NCT04904549	US	37,430	26 May 2021–13 January 2023	Occurrence of symptomatic COVID-19, presence of injection site or systemic reactions, non-serious UnSoAEs, immediate AEs, MAAEs, SAEs, AESIs, and virologically confirmed SARS-CoV-2 infections and/or symptomatic COVID-19
10	Inactivated (Vero Cells)	Shenzhen Kangtai Biological Products Co., Ltd.	Inactivated virus	China	3	NCT04852705	China	28,000	May 2021–November 2022	Incidence density of symptomatic COVID-19 cases
11	FINLAY-FR-2 anti-SARS-CoV-2 Vaccine	Instituto Finlay de Vacunas	Protein subunit	Cuba	3	IFV/COR/09	Cuba	44,010	N/A	Virologically confirmed symptomatic COVID-19 infection
12	EpiVacCorona	FBRI	Protein subunit	Russia	3	NCT04780035	Russia	3000	18 November 2020–September 2021	The proportion of vaccinated volunteers with no laboratory confirmed symptoms caused by SARS-CoV-2, within 6 months post vaccination versus placebo, the prophylactic efficacy of the vaccine
13	Recombinant (Sf9 cell)	West China Hospital	Protein subunit	China	3	NCT04904471	China	40,000	1 June 2021–31 December 2022	Virologically confirmed (polymerase chain reaction [PCR] positive) symptomatic COVID-19 cases at first appearance, regardless of severity; incidence of SAEs, AESIs, MAAEs, SoAEs, UnSoAEs
14	mRNA vaccine (ARCoV)	Academy of Military Science (AMS), Walvax Biotechnology	RNA based vaccine	China	3	NCT04847102	N/A	28,000	28 May 2021–30 May 2023	Incidence rate (person-year) of COVID-19 cases, AEs, and SAEs
15	CIGB-66	Center for Genetic Engineering and Biotechnology (CIGB)	Protein subunit	Cuba	3	RPCEC00000359	Cuba	48,000	N/A	Vaccine efficacy (number of symptomatic COVID-19 subjects with no evidence of previous exposure to viral infection)
16	VLA2001	Valneva	Inactivated virus	France	3	NCT04864561	UK	4000	26 April 2021–30 June 2022	Immune response measured after completion of a 2-dose immunization schedule, as determined by the GMT of SARS-CoV-2-specific neutralizing antibodies, frequency and severity of any AEs
17	Nanocovax	Nanogen Pharmaceutical Biotechnology	Protein Subunit	Viet Nam	3	NCT04922788	Viet Nam	13,000	7 June 2021–7 August 2022	Participants who experience a first episode of virologically confirmed case of COVID-19; any severity SAEs, MAAEs; geometric mean of anti-S IgG concentrations; geometric mean of SARS-CoV-2 serum neutralizing titres by plaque reduction neutralization test (PRNT)
18	ERUCOV-VAC (Turkovac)	Erciyes University	Inactivated virus	Turkey	3	NCT04942405	Turkey	40,800	21 June 2021–31 March 2023	Protection indexes of two vaccine doses for symptomatic COVID-19, 2 weeks after the second dose of vaccination
19	ARCT-154	Arcturus Therapeutics Inc	RNA based vaccine	US	3	ISRCTN15779782	Switzerland	N/A	3 August 2021–1 September 2023	Percentage of participants with virologically confirmed COVID-19
20	INO-4800	Inovio Pharmaceuticals	DNA based vaccine	US	3	ISRCTN15779782	Switzerland	N/A	3 August 2021–1 September 2023	Percentage of participants with virologically confirmed COVID-19
21	SCB-2019	Clover Biopharmaceuticals/GSK/Dynavax	Protein subunit	Australia	3	NCT05012787	South Africa, Ukraine	300	13 September 2021–16 December 2022	Participants with AEs, UnSoAEs, SAEs, MAAEs, AESIs, any confirmed relapse of immune-mediated disease
22	CoVLP	Medicago	Virus like particle	Canada	3	NCT04636697	Canada, US	900	22 November 2021–31 May 2022	GMTs of the three vaccine lots
23	COVAX-19^®^	Vaxine Pty.Ltd/Cinnagen Co.	Protein Subunit	Australia	2	IRCT20150303021315N24	Iran	16,876	N/A	Evaluation of COVID-19 incidence
24	DelNS1-2019-nCoV-RBD-OPT1	The University of Hong Kong and Xiamen University	Viral vector (Replicating)	Hong Kong	2	ChiCTR2100051391	Hong Kong	N/A	N/A	N/A
25	BECOV2	Biological E Limited	Protein subunit	India	3	CTRI/2021/08/036074	India	2140	N/A	Immune response measured after completion of 2-dose immunization schedule, as determined by GMT/C
26	GBP510	SK Bioscience Co., Ltd. and CEPI	Protein subunit	South Korea	3	NCT05007951	South Korea	3990	30 August 2021–September 2022	GMT of SARS-CoV-2 neutralizing antibody
27	COVI-VAC	Codagenix Inc	Live-Attenuated	US	3	ISRCTN15779782	Switzerland	N/A	3 August 2021–1 September 2023	Percentage of participants with virologically confirmed COVID-19
28	Razi Cov Pars	Razi Vaccine and Serum Research Institute	Protein subunit	Iran	2	IRCT20210206050259N3	Iran	41,128	N/A	Occurrence of confirmed symptomatic COVID-19 disease two weeks after the second vaccine dose
29	AG0301-COVID19	AnGes + Takarabio + Osaka University	DNA based vaccine	Japan	2/3	NCT04655625	Japan	500	23 November 2020–31 March 2022	Incidence of treatment-emergent AEs; Immunogenicity
30	GRAd-COV2	ReiThera	Viral vector (non-replicating)	Italy	2/3	NCT04791423	Italy	10,300	15 March 2021–30 April 2022	Participants with symptomatic laboratory confirmed COVID-19, incidence of AEs, SAEs, MAAEs, and AESIs, local and systemic SoAEs, post-treatment GMTs and GMFRs in SARS-CoV-2 S and/or RBD antibodies
31	UB-612	Vaxxinity	Protein subunit	US	2/3	NCT04683224	N/A	7320	1 February 2021–22 March 2023	The incidence of local reactions solicited systemic events, AEs, MAAEs, SAEs and AESIs, change in safety chemistry and hematology blood lab values for assessment of risk in Phase 3, prevention of SARS-CoV-2 infection in adults, change after second dose through to the end of study in antibody titres
32	GX-19	Genexine Consortium	DNA based vaccine	South Korea	2/3	NCT05067946	N/A	14,000	October 2021–October 2023	First occurrence of COVID-19 at least 14 days after the second vaccination. Incidence of SoAEs, UnSoAEs, SAEs
33	rVSV-SARS-CoV-2-S Vaccine	Israel Institute for Biological Research	Viral vector (Replicating)	Israel	2/3	NCT04990466	Israel	20,000	30 September 2021–28 February 2022	Prevention of Serology-confirmed SARS-CoV-2 infection
34	COVIran Barekat	Shifa Pharmed Industrial Co	Inactivated virus	Iran	2/3	IRCT20201202049567N3	Iran	20,000	N/A	Vaccine efficacy of Shifa-Pharmed inactivated SARS-CoV-2 vaccine
35	ReCOV	Jiangsu Rec-Biotechnology Co Ltd.	Protein subunit	China	2/3	NCT05084989	N/A	20,301	31 December 2021–31 December 2021	Number of Participants with Occurrence of COVID-19 cases, AEs, SAEs and AESIs
36	mRNA-1273.211	ModernaTX.Inc	RNA based vaccine	US	2/3	NCT04927065	US	896	24 March 2021–22 December 2021	Vaccine efficacy against SARS-CoV-2 infection; effect of vaccine on peak nasal viral load
37	AZD2816	AstraZeneca and The University of Oxford	Viral vector (non-replicating)	UK	2/3	NCT04973449	UK	2475	27 June 2021–15 June 2022	Safety and tolerability of 1 dose of AZD2816 in seronegative participants previously vaccinated with AZD1222, and 2 doses in unvaccinated seronegative participants
38	SCTV01C	Sinocelltech Ltd.	Protein Subunit	China	2/3	NCT05043311	N/A	12,420	30 October 2021–1 October 2022	The incidence of COVID-19 infections
39	FINLAY-FR-1	Instituto Finlay de Vacunas Cuba	Protein subunit	Cuba	2	IFV/COR/04	Cuba	676	13 August 2020–11 January 2021	SAEs, titre of specific anti-RBD IgG antibodies at baseline and 14, 28 and 56 days
40	LUNAR-COV19/ARCT-021	Arcturus Therapeutics Inc	RNA based vaccine	US	2	NCT04668339	Singapore, US	600	7 January 2021–30 April 2022	Local and systemic SoAEs, AEs, SAEs, MAAEs, new onset of chronic disease, abnormal chemistry and hematology values; GMT and GMFR of neutralizing antibody
41	VXA-CoV2-1	Vaxart	Viral vector (non-replicating)	US	2	NCT05067933	US	896	October 2020–June 2023	Rate of UnSoAEs, frequency of SAEs and MAAEs
42	Dendritic cell vaccine AV-COVID-19	Aivita Biomedical, Inc + Ministry of Health Republic of Indonesia	Viral vector (Replicating) + APC	US	2	NCT05007496	Indonesia	145	April 2021–May 2021	Efficacy based on T-cell-induced immune response
43	MRT5500	Sanofi Pasteur	RNA based vaccine	US	2	NCT04798027	US	333	12 March 2021–July 2022	Presence of immediate AEs, solicited injection site reactions and systemic reactions, UnSoAEs, MAAEs, and AESIs; Presence of out-of-range biological test results, neutralizing antibody titre, seroconversion
44	SARS-CoV-2 VLP Vaccine	The Scientific and Technological Research Council of Turkey	Virus like particle	Turkey	2	NCT04962893	Turkey	330	26 June 2021–September 2022	Comparison of efficacy, specific IgG, neutralizing antibody, and cellular immune response
45	Recombinant SARS-CoV-2 Fusion Protein Vaccine (V-01)	Guangdong Provincial Center for Disease Control and Prevention	Protein subunit	China	2	ChiCTR2100045107	China	880	28 March 2021–30 July 2022	Positive conversion rate of serum anti-SARS-CoV-2 RBD protein antibody, and its GMT and GMI; positive conversion rate of serum anti-SARS-CoV-2 neutralizing antibody, and its GMI
46	SCB-2020S	Clover Biopharmaceuticals AUS Pty Ltd.	Protein subunit	Australia	2	NCT04950751	N/A	150	August 2021–April 2020	GMT and GMFR of SARS-CoV-2 neutralising antibodies to B.1.351 variant, proportion of subjects achieving seroconversion of SARS-CoV-2 neutralising antibodies to B.1.351 variant
47	SC-Ad6-1	Tetherex Pharmaceuticals Corporation	Viral vector (non-replicating)	US	2	NCT05077267	Germany	210	19 August 2021–1 February 2024	SARS-CoV-2 neutralizing antibody titers
48	Recombinant RBD Protein Vaccine	Bagheiat-allah University of Medical Sciences	Protein subunit	Iran	2	IRCT20210620051639N2	Iran	300	N/A	IgG antibody against Receptor Binding Domain (RBD) protein
49	KBP-201	Kentucky Bioprocessing	Protein subunit	US	1/2	NCT04473690	US	180	31 July 2021–31 July 2022	Solicited administration site reactions and systemic events
50	RBD SARS-CoV-2 HBsAg VLP	SpyBiotech + Serum Institute of India	Virus like particle	UK	1/2	ACTRN12620000817943	Australia	280	N/A	To assess the immune response, safety and reactogenicity, of a two-dose schedule of two dose amounts of RBD SARS-CoV-2 HBsAg VLP vaccine as compared with two-dose administration of placebo
51	IMP CoVac-1	University Hospital Tuebingen	Protein subunit	Germany	1/2	NCT04954469	Germany	68	30 June 2021–31 March 2022	Safety- Eastern Cooperative Oncology Group (ECOG) status, vital signs, blood chemistry and coagulation, and hematology
52	LV-SMENP-DC	Shenzhen Geno-Immune Medical Institute	Viral vector (non-replicating) + APC	China	1/2	NCT04276896	China	100	24 March 2020–31 December 2024	Clinical improvement based on the 7-point scale, lower Murray lung injury score
53	hAd5-S+N bivalent vaccine	ImmunityBio Inc	Viral vector (non-replicating)	US	1/2	NCT04843722	US	540	May 2021–August 2022	Efficacy: percent of subjects that show an increase in N-reactive T cells
54	CIGB-669	Center for Genetic Engineering and Biotechnology (CIGB)	Protein subunit	Cuba	1/2	RPCEC00000345	Cuba	88	N/A	Safety: occurrence and intensity of AEs, subjects with seroconversion of anti-RBD IgG antibodies to SARS-CoV-2
55	AdCLD-CoV19	Cellid Co., Ltd.	Viral vector (non-replicating)	South Korea	1/2	NCT04666012	South Korea	150	29 December 2020–April 2022	Incidence of SoAEs and UnSoAEs
56	GLS-5310	GeneOne Life Science Inc	DNA based vaccine	South Korea	1/2	NCT04673149	South Korea	345	23 December 2020–31 December 2022	Incidence of AEs; GMT of antigen-specific binding antibody titres
57	S-268019	Shionogi	Protein subunit	Japan	1/2	jRCT2051200092	Japan	214	N/A	AEs, adverse reactions, SAEs, local and systemic reactogenicity SoAEs, GMT of SARS-CoV-2 neutralizing antibody
58	SARS-CoV-2-RBD-Fc-fusion protein	University Medical Center Groningen + Akston Biosciences Inc.	Protein subunit	The Netherlands	1/2	NCT04681092	The Netherlands	130	12 April 2021–30 June 2021	Safety / Tolerability (35 days)
59	COVAC-1 and COVAC-2	University of Saskatchewan	Protein subunit	Canada	1/2	NCT04702178	Canada	108	10 February 2021–February 2023	AEs during 28 days after each injection
60	COVID-eVax	Takis + Rottapharm Biotech	DNA based vaccine	Italy	1/2	NCT04788459	Italy	160	25 February 2021–June 2022	Local and systemic SoAEs, UnSoAEs, quantitative antibody titres, binding to the specific SARS-CoV-2 antigen, SARS-CoV-2 neutralizing antibody titre, change from baseline in antigen-specific cellular immune responses to SARS-CoV-2, percentage of subjects who seroconverted
61	Inactivated (NDV based) chimeric vaccine	Mahidol University and Government Pharmaceutical Organization	Inactivated virus	Thailand	1/2	NCT04764422	Thailand	460	20 March 2021–April 2023	Frequency of reportable local and systemic SoAEs after each vaccination; measurement of changes in hemoglobin, white blood cells, platelet count, creatinine, AST, ALT, bilirubin
62	VBI-2902a	VBI Vaccines Inc	Virus like particle	Canada	1/2	NCT04773665	Canada	780	15 March 2021–June 2022	Rate and severity of local and systemic SoAEs, UnSoAEs, MAAEs, SAEs, and laboratory abnormalities; AEs leading to discontinuation of study vaccination
63	EuCorVac-19	POP Biotechnologies and EuBiologics Co Ltd.	Protein subunit	South Korea	1/2	NCT04783311	South Korea	280	23 February 2021–January 2023	Immediate AEs, local and systemic AEs, UnSoAEs, SAEs, AESIs.
64	DS-5670a	Daiichi Sankyo Co Ltd.	RNA based vaccine	Japan	1/2	NCT04821674	Japan	152	15 March 2021–31 December 2022	Number of participants reporting treatment-emergent AEs, local and systemic AEs, and SAEs; GMT, GMFR, and SCR of SARS-CoV-2 specific neutralizing antibody
65	COVIVAC	Institute of Vaccines and Medical Biologicals	Viral vector (non-replicating)	Viet Nam	1/2	NCT04830800	Viet Nam	420	10 March 2021–30 September 2022	Number and severity of local and systemic SoAEs, UnSoAEs, SAEs, MAAEs, AESIs, and clinically significant hematological and biochemical measurements
66	Recombinant SARS-CoV-2 Vaccine (CHO Cell)	National Vaccine and Serum Institute	Protein subunit	China	1/2	NCT04869592	China	3580	25 April 2021–25 October 2022	Incidence and severity of any adverse reactions/events and abnormal blood biochemistry, blood routine, blood coagulation function and urine routine, SAEs, AESIs, GMT of SARS-CoV-2 neutralizing antibody
67	EXG-5003	Elixirgen Therapeutics Inc	RNA based vaccine	Japan	1/2	NCT04863131	Japan	60	28 April 2021–31 January 2023	Number of participants reporting local and systemic AEs
68	KD-414	KM Biologics Co Ltd.	Inactivated virus	Japan	1/2	jRCT2071200106	Japan	210	N/A	Safety: all adverse events and immunogenicity: neutralizing antibody conversion rate against SARS-CoV-2
69	MVA vector expressing stabilized S Protein	German Centre for Infection Research	Viral vector (non-replicating)	Germany	1/2	NCT04895449	Germany	240	1 June 2021–1 March 2022	Percentage of participants experiencing solicited local or systemic reactogenicity as defined by the study protocol
70	QazCovac	Research Institute for Biological Safety Problem	Protein subunit	Kazakhstan	1/2	NCT04930003	Kazakhstan	244	15 June 2021–December 2021	Frequency of AEs for up to 7 and 21 days after immunization. The proportion of volunteers with increased levels of the immune response of specific neutralizing antibody titres using ELISA following the vaccination, compared with placebo
71	AG0302-COVID19	AnGes, Inc/Osaka University	DNA based vaccine	Japan	1/2	NCT04993586	Japan	400	29 July 2021–31 December 2021	Incidence of Treatment-Emergent AEs, immunogenicity
72	Hipra	Laboratorios Hipra, S.A.	Protein subunit	Spain	1/2	NCT05007509	Spain	30	16 August 2021–September 2022	Local and systemic SoAEs and UnSoAEs
73	Versamune-CoV-2FC vaccine	Farmacore Biotecnologia Ltd.a	Protein subunit	Brazil	1/2	NCT05016934	N/A	N/A	1 November 2021–20 April 2022	Frequency and severity of local and systemic AEs and AESIs.
74	ARCT-165	Arcturus Therapeutics Inc	RNA based vaccine	US	1/2	NCT05037097	Singapore, US	72	30 August 2021–March 2023	Local and systemic SoAEs, AEs, SAEs, MAAEs, new onset of chronic disease, abnormal chemistry and hematology values; GMT and GMFR of neutralizing antibody
75	ARCT-021	Arcturus Therapeutics Inc	RNA based vaccine	US	1/2	NCT05037097	Singapore, US	72	30 August 2021–March 2023	Local and systemic SoAEs, AEs, SAEs, MAAEs, new onset of chronic disease, abnormal chemistry and hematology values; GMT and GMFR of neutralizing antibody
76	SII B.1.351, a monovalent (Beta) variant	Novavax	Protein subunit	US	1/2	NCT05029856	Australia	240	February 2022–August 2022	MN50 GMTs to the SARS-CoV-2 B.1.351 (Beta) and B.1.617.2 (Delta), expressed as GMT and SCRs/SRRs. Local and systemic SoAEs, UnSoAEs, MAAEs
77	SII Bivalent: (ancestral strain and (Beta) variant)	Novavax	Protein subunit	US	1/2	NCT05029856	Australia	240	February 2022–August 2022	MN50 GMTs to the SARS-CoV-2 B.1.351 (Beta) and B.1.617.2 (Delta), expressed as GMT and SCRs/SRRs. Local and systemic SoAEs, UnSoAEs, MAAEs
78	SII B.1.617.2, monovalent (Delta) variant	Novavax	Protein subunit	US	1/2	NCT05029856	Australia	240	February 2022–August 2022	MN50 GMTs to the SARS-CoV-2 B.1.351 (Beta) and B.1.617.2 (Delta), expressed as GMT and SCRs/SRRs. Local and systemic SoAEs, UnSoAEs, MAAEs
79	AAV5-RBD-S vaccine (BCD-250)	Biocad	Viral vector (non-replicating)	Russia	1/2	NCT05037188	Russia	160	10 August 2021–December 2022	Percentage of subjects with ≥ 4 fold rise of serum SARS-CoV-2-specific IgG titer from baseline
80	CoviVac	Chumakov Federal Scientific Center for Research and Development of Immune-and-Biological Products	Inactivated virus	Russia	1/2	NCT05046548	Russia	400	3 October 2020–1 October 2021	Geometric mean titer (GMT)
81	VB10.2129, encoding RBD	Vaccibody AS	DNA based vaccine	Norway	1/2	NCT05069623	Norway	160	27 October 2021–October 2023	Local and systemic SoAEs, UnSoAEs, SAEs
82	VB10.2210	Vaccibody AS	DNA based vaccine	Norway	1/2	NCT05069623	Norway	160	27 October 2021–October 2023	Local and systemic SoAEs, UnSoAEs, SAEs
83	SARS-CoV-2 Protein Subunit Recombinant Vaccine	Biofarma	Protein subunit	Indonesia	1/2	NCT05067894	Indonesia	780	November 2021–March 2022	Safety (phase I) and immunogenicity (phase II) of the SARS-CoV-2 protein subunit recombinant vaccine.
84	MVA-SARS-2-S	University of Munich	Viral vector (non-replicating)	Germany	1	NCT04569383	Germany	30	October 2020–May 2021	Percentage of participants experiencing solicited local or systemic reactogenicity
85	LNP-nCoVsaRNA	Imperial College London	RNA based vaccine	UK	1	ISRCTN17072692	UK	320	1 April 2020–31 July 2021	Solicited local injection site reactions, solicited systemic and laboratory reactions, UnSoAEs, SAEs, titres of neutralizing antibody and IgG
86	Covid-19/aAPC	Shenzhen Geno-Immune Medical Institute	Viral vector (Replicating)	China	1	NCT04299724	China	100	15 February 2020–31 December 2024	Frequency of vaccine events and serious vaccine events, proportion of subjects with positive T cell response
87	AdimrSC-2f	Adimmune Corporation	Protein subunit	Taiwan	1	NCT04522089	Taiwan	70	20 August 2020–20 March 2021	SoAEs and incidence of abnormal laboratory tests results
88	Covigenix VAX-001	Entos Pharmaceuticals Inc	DNA based vaccine	Canada	1	NCT04591184	Canada	72	7 April 2021–August 2022	Safety of a 2-dose regimen of VAX-001 when doses are given 14 days apart, Mean change from baseline in safety laboratory measures, frequency of treatment-emergent SAEs throughout the study and up to 12 months post-second dose
89	CORVax	Providence Health & Services	DNA based vaccine	US	1	NCT04627675	US	36	30 December 2020–May 2022	Toxicity and MAAEs at various time frames
90	ChulaCov19	Chulalongkorn University	RNA based vaccine	Thailand	1	NCT04566276	Thailand	96	January 2021–June 2021	Frequency and grade of AEs, reportable local and systemic SoAEs
91	bacTRL-Spike	Symvivo corporation	DNA based vaccine	Canada	1	NCT04334980	Australia	24	2 November 2020–28 February 2022	Frequency of adverse events up to 12 months post-vaccination
92	COH04S1	City of Hope Medical Center	Viral vector (non-replicating)	US	1	NCT04639466	US	129	11 December 2020–10 November 2022	Incidence of adverse events for up to 365 days
93	MF59	The University of Queensland	Protein Subunit	Australia	1	NCT04495933	Australia	216	13 July 2020–8 November 2021	Local and systemic SoAEs, UnSoAEs, SAEs, GMT of the serum antibody and Nab response
94	COVIGEN	The University of Sydney Bionet Co., Ltd.	DNA based vaccine	Australia	1	NCT04742842	Australia	150	15 February 2021–31 December 2022	Frequency of solicited local and systemic reactogenicity AEs, UnSoAEs, SAEs, MAAEs, change in safety laboratory values from baseline
95	BBV154	Bharat Biotech	Viral vector (non-replicating)	India	1	NCT04751682	India	175	1 March 2021–30 November 2021	Incidence of immediate AEs, local and systemic SoAEs, SAEs, and UnSoAEs
96	PTX-COVID19-B	Providence Therapeutics Holdings Inc	RNA based vaccine	Canada	1	NCT04765436	Canada	60	14 January 2021–14 February 2022	Occurrence of AEs after each vaccination, assessments of AEs (days 1-42) and safety (days 1-395), immunogenicity analysis
97	CoV2 SAM (LNP)	GlaxoSmithKline	RNA based vaccine	UK	1	NCT04758962	US	40	15 February 2021–9 May 2022	Participants with at least 1 solicited administration site event and solicited systemic event, during 7-day follow-up period, with any UnSoAEs during 30-day follow-up period after each vaccination, and with any hematological and biochemical laboratory abnormality at screening, day 1, 2, 8, 31, 32, and 38.
98	NBP2001	SK Bioscience Co Ltd.	Protein subunit	South Korea	1	NCT04760743	South Korea	50	17 December 2020–April 2022	Occurrence of immediate systemic reactions, local and systemic SoAEs, UnSoAEs, SAEs, MAAEs, and AESIs, GMT and GMFR of IgG and neutralizing antibody to the SARS-CoV-2
99	ChAdV68-S and AM-LNP-S	Gritstone Oncology	Viral vector (non-replicating)	US	1	NCT04776317	US	130	25 March 2021–19 September 2022	Frequency by grade of solicited local reactogenicity AEs, solicited systemic reactogenicity AEs, UnSoAEs, AESIs, clinical safety laboratory AEs by severity grade and SAEs
100	SpFN COVID-19 Vaccine	Walter Reed Army Institute of Research	Protein subunit	US	1	NCT04784767	US	72	5 April 2021–30 October 2023	Participants with local and systemic reactions, incidents of treatment-adverse events as assessed by FDA toxicity grading scale, and participants with humoral immune response
101	FAKHRAVAC (MIVAC)	Organization of Defensive Innovation and Research	Inactivated virus	Iran	1	IRCT20210206050259N1	Iran	135	N/A	Abnormal vital signs and anaphylactic reactions immediately after vaccination, local and systemic AEs within the first week post-vaccination, abnormal laboratory findings
102	MV-014-212	Meissa Vaccines Inc	Live-Attenuated	US	1	NCT04798001	US	130	12 April 2021–31 October 2022	SoAEs, UnSoAEs, SAEs, MAAEs, and change in serum neutralizing antibody titres against vaccine-encoded SARS-CoV-2 S protein
103	Koçak-19 Inaktif Adjuvanlı COVID-19 Vaccine	Kocak Farma	Inactivatedviral vecviral virus	Turkey	1	NCT04838080	Turkey	38	19 March 2021–20 October 2021	AEs, from day 0 until the end of follow up period of 6 months
104	ABNCoV2	Radboud University	Virus like particle	The Netherlands	1	NCT04839146	The Netherlands	42	11 March 2021–20 December 2021	Safety: possibly related Grade 3 AEs and SAEs, Immunogenicity endpoint: concentration of ABNCoV2-specific antibodies
105	HDT-301	SENAI CIMATEC	RNA based vaccine	Brazil	1	NCT04844268	N/A	78	May 2021–July 2022	Safety and tolerability of two doses of HDT-1 vaccine (day 1, 29 and 57)
106	Adjuvanted Inactivated Vaccine	The Scientific and Technological Research Council of Turkey	Inactivated virus	Turkey	1	NCT04866069	Turkey	50	25 April 2021–April 2022	AAEs, local and systemic SoAEs and UnSoAEs
107	mRNA-1283	ModernaTX, Inc	RNA based vaccine	US	1	NCT04813796	US	125	11 March 2021–13 April 2022	Number of participants with solicited local and systemic reactogenicity Adverse Reactions (ARs), UnSoAEs, and MAAEs, AESIs, and SAEs
108	Recombinant NDV Vectored Vaccine	Laboratorio Avi-Mex	Inactivated virus	Mexico	1	NCT04871737	Mexico	90	20 May 2021–June 2022	AEs, pregnancy test, urinalysis, oxygen saturation
109	mRNACOVID-19 Vaccine	Stemirna Therapeutics Co Ltd. and Shanghai East Hospital	RNA based vaccine	China	1	ChiCTR2100045984	China	30	25 March 2021–25 May 2022	Safety and immunogenicity
110	CoVepiT	OSE Immunotherapeutic	Protein subunit	Belgium	1	NCT04885361	Belgium	48	26 May 2021–31 March 2022	The incidence of solicited local and systemic reactogenicity signs and symptoms, UnSoAEs, SAEs, AESIs, subjects with significantly increased CD8+ T cells responding to SARS-CoV-2
111	CoV2-OGEN1	USSF/Vaxform	Protein subunit	US	1	NCT04893512	N/A	45	7 June 2021–15 September 2022	Safety evaluation of 2-dose vaccination schedule of orally administered CoV2-OGEN1 by following local and systemic adverse events
112	LNP-nCOV saRNA-02 Vaccine	MRC/UVRI and LSHTM Uganda Research Unit	RNA based vaccine	Uganda	1	NCT04934111	Uganda	42	September 2021–August 2022	Participant with solicited local injection site and systemic reactions, UnSoAEs, SAEs, titre of neutralizing antibody and IgG
113	Baiya SARS-CoV-2 Vax 1 Vaccine	Baiya Phytopharm Co Ltd.	Protein subunit	Thailand	1	NCT04953078	N/A	96	September 2021–November 2022	Frequency and grade SoAEs and AEs, change in blood pressure, pulse rate, respiratory rate, and physical condition, safety laboratory value
114	PIV5-SARS CoV-2	CyanVac LLC	Viral vector (non-replicating)	US	1	NCT04954287	US	80	July 2021–November 2022	SoAEs, UnSoAEs
115	202-CoV	Shanghai Zerun Biotechnology, Walvax Biotechnology	Protein subunit	China	1	NCT04982068	China	144	12 July 2021–October 2022	SoAEs, UnSoAEs
116	COVIDITY	Scancell Ltd.	DNA based vaccine	UK	1	NCT05047445	South Africa	40	30 September 2021–June 2022	Safety and tolerability of COVIDITY as assessed by AEs, vital signs, and physical examination
117	PIKA-adjuvanted vaccine	Yisheng Biopharma	Protein subunit	Singapore	1	ACTRN12621001009808	Australia, New Zealand	45	24 September 2021–13 June 2022	To assess the safety and tolerability of the PIKA COVID-19 vaccine by monitoring AEs and clinical laboratory tests
118	SARS-CoV-2 DNA vaccine	The University of Hong Kong; Immuno Cure 3 Limited	DNA based vaccine	Hong Kong	1	NCT05102643	Hong Kong	30	November 2021–December 2022	Reactogenicity and AEs
119	Ad5-triCoV/Mac or ChAd-triCoV/Mac	McMaster University	Viral vector (non-replicating)	Canada	1	NCT05094609	Canada	30	9 November 2021–30 June 2023	Number of participants reporting AEs and severity of AEs following vaccination
120	T-cell priming specific peptides on a gold nanoparticle	Emergex Vaccines	Protein subunit	Switzerland	1	NCT05113862	Switzerland	26	December 2021–November 2022	Local and systemic SoAEs, UnSoAEs, SAEs, AESIs, SAEs, MAAEs, AESIs. GMT and GMFR of Anti-SAS-CoV-2 RBD IgG and neutralizing anti-SARS-CoV-2
121	IN-B009	HK inno.N Corporation	Protein subunit	South Korea	1	NCT05113849	South Korea	40	16 September 2021–February 2023	Occurrence of IAR, local and systemic AE, UnSoAE,

MAAEs: Medically Attended Adverse Events, NOCMCs: New-onset Chronic Medical Conditions, SAEs: Serious adverse events, SCR: Seroconversion Rate, SRR: Seroresponse Rate, SoAEs: The solicited adverse events, UnSoAEs: The unsolicited adverse events, TEAE: Treatment Emergent Adverse Event, IAR: Immediate Adverse Reaction.
